# Internal transcribed spacer 2 barcode: a good tool for identifying Acanthopanacis cortex

**DOI:** 10.3389/fpls.2015.00840

**Published:** 2015-10-08

**Authors:** Sha Zhao, Xiaochen Chen, Jingyuan Song, Xiaohui Pang, Shilin Chen

**Affiliations:** ^1^Institute of Medicinal Plant Development, Chinese Academy of Medical Sciences and Peking Union Medical CollegeBeijing, China; ^2^Institute of Chinese Materia Medica, China Academy of Chinese Medical SciencesBeijing, China

**Keywords:** Acanthopanacis cortex, DNA barcoding, herbal medicine, identification, ITS2

## Abstract

Acanthopanacis cortex has been used in clinical applications for a long time. Considering some historical and geographical factors, Acanthopanacis cortex is easily confused with other herbs in medicine markets, thereby causing potential safety issues. In this study, we used the internal transcribed spacer 2 (ITS2) barcode to identify 69 samples belonging to six species, including Acanthopanacis cortex and its adulterants. The nearest distance, single-nucleotide polymorphisms (SNPs), and neighbor-joining (NJ) tree methods were used to evaluate the identification ability of the ITS2 barcode. According to the kimura-2-parameter model, the intraspecific distance of *Eleutherococcus nodiflorus* ITS2 sequences ranged from 0 to 0.0132. The minimum interspecific distance between *E. nodiflorus* and *E. giraldii* was 0.0221, which was larger than the maximum intraspecific distance of *E. nodiflorus*. Three stable SNPs in ITS2 can be used to distinguish Acanthopanacis cortex and its closely related species. The NJ tree indicated that the Acanthopanacis cortex samples clustered into one clade, which can be distinguished clearly from the adulterants of this herb. A secondary structure of ITS2 provided another dimensionality to identify species. In conclusion, the ITS2 barcode effectively identifies Acanthopanacis cortex, and DNA barcoding is a convenient tool for medicine market supervision.

## Introduction

Acanthopanacis cortex, a traditional Chinese medicine known as “wujiapi,” is derived from the dried root barks of *Eleutherococcus nodiflorus* (Dunn) S.Y. Hu (State Pharmacopoeia Committee, 2010). It is commonly used to expel wind-dampness, tonify liver and kidneys, and strengthen muscles and bones, in accordance with Chinese medicine theory (State Pharmacopoeia Committee, 2010). In China, *Acanthopanax gracilistylus* wine, which is made of Acanthopanacis cortex and other herbs soaked in liquor, is popularly used as a health supplement product to treat rheumatic arthritis because of its clinical effects. To date, Acanthopanax cortex has been reported to exhibit anti-inflammatory and potential antitumor activities, as well as demonstrate treatment effects for bone diseases (Shan et al., 1999, 2000; Zhang et al., 2012). As a candidate therapy, Acanthopanax cortex particularly exerts anti-inflammatory and protective effects on the liver, thereby showing potential treatment for fulminant hepatitis (Zhang et al., 2011). Given the curative effects of Acanthopanax cortex, this herb has been widely used in clinical applications and attracted increasing research attention.

**Figure 1:**
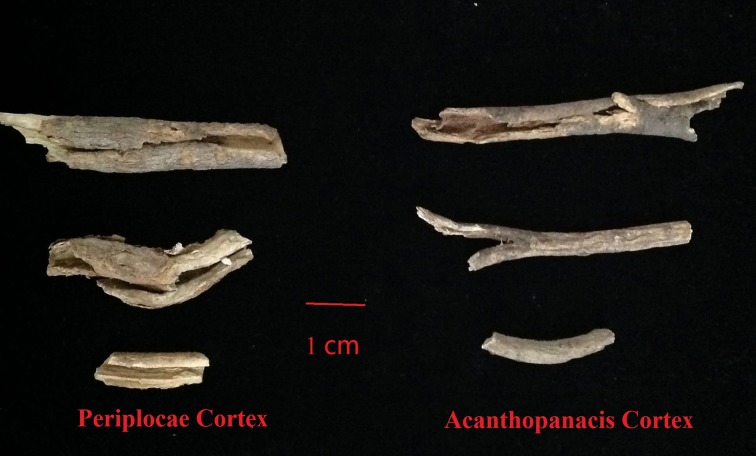
**Samples of Acanthopanacis cortex and Periplocae cortex collected from medicine markets in China**.

However, Acanthopanacis cortex is easily confused with other herbs in medicine markets, thereby causing potential safety issues (Wu et al., 2014). The original species of Acanthopanacis cortex is *E. nodiflorus*. The genus *Eleutherococcus* Maxim. consists of 18 species that are widely distributed throughout China (Flora of China Editorial Committee, 2007). Considering some historical and geographical factors, some closely related species of *E. nodiflorus* are commonly used as local herbs in various places. For example, *E. giraldii* (Harms) Nakai, a Tibetan medicine distributed in northwest and southwest China, is mainly used as Acanthopanacis cortex in Sichuan Province (Wang et al., 2005). The root bark from *E. sessiliflorus* (Rupr. and Maxim.) S.Y. Hu is considered one of the sources of Acanthopanacis cortex in northeast China (Song et al., 2012). The wide application of alternatives may influence the efficacy of clinical medication. Furthermore, Acanthopanacis cortex is frequently adulterated by Periplocae cortex, known as “xiangjiapi,” which is the root cortex of *Periploca sepium* Bge (Guo et al., 2013; Wu et al., 2014). Both herbs share similar morphological characteristics and name (Figure 1), but the functions and efficacies of these herbs are not quite the same. Periplocae cortex contains toxic components that may cause poisoning and has been implicated in various clinical accidents (Liang et al., 2014). *Acanthopanax gracilistylus* wine poisoning has been reportedly caused by Periplocae cortex substitution for Acanthopanacis cortex. Therefore, Acanthopanacis cortex should be correctly identified. However, identifying cortex herbs using traditional identification methods is difficult, especially when the cortex is dried and sliced (Sun and Chen, 2013; Huang et al., 2014; Techen et al., 2014). Thus, a good tool is needed to identify Acanthopanacis cortex accurately.

DNA barcoding technique uses short DNA sequences to identify species (Hebert et al., 2003), which makes it possible to correctly identify Acanthopanacis cortex. DNA barcoding has been widely used to identify herbs in recent years. Xiang et al. (2013) accurately identified 131 *Ophiocordyceps sinensis* (Berk.) G. H. Sung samples, as well as 12 common counterfeits and its closely related species by using DNA barcoding. A fast SNP identification based on DNA barcoding was used to identify the genus *Panax* L. with its closely related species, as well as the mixed powder *Panax ginseng* C. A. Mey. and *P. quinquefolius* L. (Chen et al., 2013). Xin et al. (2013) used ITS2 barcode to successfully distinguish *Lycium barbarum* L., *L. chinense* Mill., and *L. ruthenicum* Murray and indicated ITS2 barcode as a powerful tool to trace Goji. Li et al. (2012) used DNA barcode and chemical profiling techniques to successfully identify Baiying (Herba Solani Lyrati) commodity and its toxic substitute Xungufeng (Herba Aristolochiae Mollissimae). Newmaster et al. (2013) used DNA barcoding to detect the North American herbal products, and suggested that the herbal industry should embrace DNA barcoding for authenticating herbal products, which would provide consumers with safe, high quality herbal products. These studies indicated that DNA barcoding is a convenient tool to identify medicinal plants, herbal medicine, and even commercial products. To ensure the clinical safety of Acanthopanacis cortex, the ITS2 barcode was sequenced and used in the present study to identify this herb and its adulterants.

## Materials and methods

### Plant materials

A total of 69 samples belonging to six species, including *E. nodiflorus* (7 cortex and 9 leaf samples), *P. sepium* (12 cortex and 3 leaf samples), *E. senticosus* Maxim. (10 root, 1 fruit, 1 stem, and 3 leaf samples), *E. giraldii* (5 cortex and 4 leaf samples), *E. sessiliflorus* (1 stem and 8 leaf samples), and *E. trifoliatus* (L.) S. Y. Hu (1 leaf sample and 4 ITS2 sequences downloaded from GenBank), were collected from medicine markets, drug stores, botanical gardens, and fields (Supplementary Table 1). Experimental samples bought from medicine markets and drug stores were already dried in silica gel, and collected from fields. The corresponding voucher samples were deposited in the herbarium of the Institute of Medicinal Plant Development, Chinese Academy of Medical Sciences, Beijing, China.

### Molecular analyses

The surfaces of the samples were first scraped off then wiped with 75% ethanol. Total genomic DNA was extracted from 20 to 40 mg dry powder of the tissues by using the Plant Genomic DNA Kit (Tiangen Biotech Co.). Polymerase chain reaction (PCR) amplification and sequencing were performed as described in previous study (Chen et al., 2010). Raw trace files were trimmed and assembled using CodonCode Aligner 4.2.7 (CodonCode Co., USA). The ITS2 region was annotated using the Hidden Markov model to remove the 5.8S and 28S sections (Keller et al., 2009). All 69 ITS2 sequences were used to calculate genetic distances and construct a neighbor-joining (NJ) tree. Additionally, 54 ITS2 sequences from *E. nodiflorus* and its closely related species (*E. senticosus, E. giraldii, E. sessiliflorus, and E. trifoliatus*) were used to detect single-nucleotide polymorphisms (SNPs). Six haplotypes of Acanthopanacis cortex and its adulterants were used to predict the secondary structure of ITS2. Genetic distances were calculated according to the kimura-2-parameter (K2P) model using MEGA 5.2.2 software (Tamura et al., 2011). SNP sites were detected using Sequencher 5.3 software (Gene Codes Co., USA). An NJ tree was constructed, and bootstrap tests were performed using 1000 replicates to identify Acanthopanacis cortex and its adulterants via MEGA 5.2.2 (Tamura et al., 2011). The ITS2 secondary structure was predicted using the ITS2 Workbench (http://its2.bioapps.biozentrum.uni-wuerzburg.de/) (Koetschan et al., 2012).

## Results

### Analysis of PCR amplification and sequence characteristics

The PCR amplification success rate of the ITS2 region was 100%. All purified PCR products were successfully sequenced, and high-quality bidirectional trace files were generated. All the obtained sequences were submitted to GenBank (Supplementary Table 1).

The PCR product sequence lengths of all 69 samples ranged from 420 to 470 bp. After annotation, the ITS2 sequence of 16 *E. nodiflorus* samples was 230 bp in length, and the average GC content was 62.4%. Four nucleotide variation sites were found in these sample sequences. Four haplotypes of ITS2 sequences were generated. The ITS2 sequence lengths of *P. sepium, E. senticosus, E. giraldii, E. sessiliflorus*, and *E. trifoliatus*, which were adulterants of Acanthopanacis cortex, were 235, 230, 230, 230, and 231 bp, respectively, after alignment; the corresponding average GC content of the above adulterants were 55.7, 62.6, 63.4, 62.2, and 63.9%. Only one and three intraspecies variation sites were found in the ITS2 regions of *E. giraldii* and *E. trifoliatus*, respectively. The ITS2 regions of *P. sepium, E. senticosus*, and *E. sessiliflorus* were highly conservative, and no intraspecies variation sites existed in these regions.

### Analysis of SNPs, inter/intra-specific distance, and NJ tree

SNP analysis allowed the successful detection and distinction of specific genetic variations, which were effectively applied in plant and animal identification (Chen et al., 2013; Engdahl et al., 2014). The said method is particularly used to analyze closely related species (Arif et al., 2010; Chen et al., 2013). A total of 54 ITS2 sequences from *E. nodiflorus* and its closely related species (*E*. *senticosus, E. giraldii, E. sessiliflorus, and E. trifoliatus*) were analyzed to detect the SNPs at an interspecific level. Variable sites in the haplotypes of the five *Eleutherococcus* species are shown in Supplementary Figure 1. Three stable variation sites at the positions of 23, 36, and 170 bp were found (Table 1), which can be used to distinguish Acanthopanacis cortex and its closely related species. All the published ITS2 sequences of *E. nodiflorus* and its closely related species were downloaded from GenBank. The three SNPs existed stably in these sequences, which further verified the above results.

**Table 1:** **Three stable SNPs in ITS2 sequences from *Eleutherococcus nodiflorus* and its closely related species**.

**Species name**	**SNP location**		
	**23 bp**	**36 bp**	**170 bp**
*E. nodiflorus*	A	C	A
*E. senticosus*	C	T	C
*E. giraldii*	C	T	C
*E. sessiliflorus*	C	T	C
*E. trifoliatus*	C	T	C

*The E. nodiflorus sequence (GenBank No. KR080395) is used as reference sequence for locating SNP sites*.

All the 69 ITS2 sequences were analyzed to calculate the genetic distances. According to the K2P model, the intraspecific distance of *E. nodiflorus* varied from 0 to 0.0132, with an average of 0.0078. The maximum intraspecific distances of *E. giraldii* and *E. trifoliatus* were 0.0044 and 0.0131, respectively. The average interspecific distance between Acanthopanacis cortex and its adulterants was 0.0510, whereas the maximum interspecific distance was 0.3818, which was attributed to *E. nodiflorus* and *P. sepium*. The minimum interspecific distance of 0.0221 between *E. nodiflorus* and *E. giraldii* was larger than the maximum intraspecific distance of *E. nodiflorus* (Table 2). Overall, the results indicated that the ITS2 barcode can correctly identify Acanthopanacis cortex and its adulterants.

**Table 2:** **Intraspecific and interspecific genetic distances between Acanthopanacis cortex and its adulterants**.

**K2P genetic distances**	**Range of genetic distances (mean)**
Intraspecific distances of *Eleutherococcus nodiflorus*	0–0.0132 (0.0078)
Intraspecific distances of *E. giraldii*	0–0.0044 (0.0017)
Intraspecific distances of *E. trifoliatus*	0–0.0131 (0.0061)
Interspecific distances between Acanthopanacis cortex and its adulterants	0.0221–0.3818 (0.0510)

**Figure 2:**
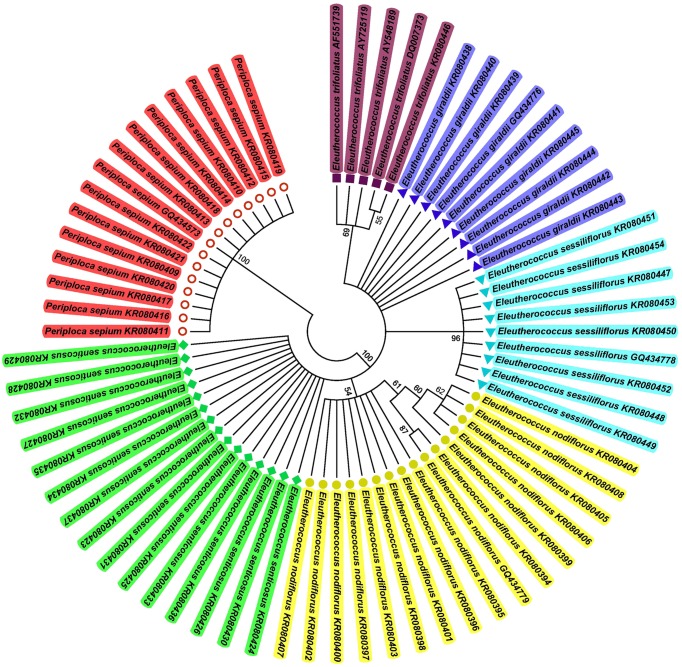
**NJ tree of Acanthopanacis cortex and its adulterants constructed with ITS2 sequences**. The bootstrap scores (1000 replicates) are shown (≥50%) for each branch.

All the 69 ITS2 sequences were used to construct the NJ tree. The findings showed that *E. nodiflorus* formed into one clade, whereas the adulterants clustered into other clades. Thus, the NJ tree can successfully distinguish Acanthopanacis cortex from its adulterants (Figure 2).

### Analysis of secondary structure

**Figure 3:**
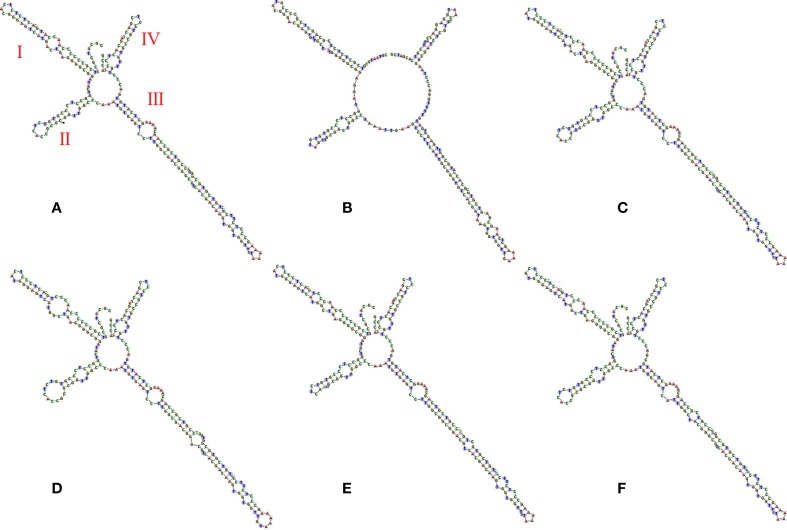
**Secondary structure of ITS2 in Acanthopanacis cortex and its adulterants**. **(A)** *Eleutherococcus nodiflorus* (GQ434779), **(B)** *Periploca sepium* (KR080415), **(C)** *E. senticosus* (KR080436), **(D)** *E. giraldii* (KR080439), **(E)** *E. sessiliflorus* (GQ434778), and **(F)** *E. trifoliatus* (KR080446).

Six haplotypes of Acanthopanacis cortex and its adulterants were used to predict the secondary structure of ITS2. All six ITS2 secondary structures exhibited four similar helices: Helix I, II, III, and IV. The secondary structures between *E. nodiflorus* and *P. sepium* showed significant differences on Helix I, II, III, and IV. The secondary structures between *E. nodiflorus* and its related species were relatively conservative, but the position, size, and number of Loops in Helix I, II, and III showed differences, which can be used as molecular morphological characteristics to identify species (Figure 3). Thus, the ITS2 secondary structure provided an auxiliary method to distinguish Acanthopanacis cortex and its adulterants.

## Discussion

### Why we selected ITS2 barcode to identify acanthopanacis cortex

The establishment of a standard DNA barcode is an important premise of DNA barcoding applications. Chen et al. (2010) analyzed more than 6600 medicinal plants and their closely related species and evaluated the identification ability of the genomic regions *psbA-trnH, matK, rbcL, rpoC1, ycf5*, internal transcribed spacer (ITS), and ITS2; they determined that the rate of successful identification with ITS2 barcode was 92.7% at the species level and suggested that the ITS2 DNA barcode represents the most suitable region to identify medicinal plants. The identification efficiency of the ITS2 barcode has been validated in several families and genera of medicinal plants (Gao et al., 2010; Pang et al., 2011; Gu et al., 2013). The China Plant BOL Group (2011) confirmed that the ITS/ITS2 regions should be incorporated into the core barcode for seed plants. Furthermore, the stability and accuracy of the ITS2 barcode have been verified in herbal medicine (Chen et al., 2013; Hou et al., 2013; Xiang et al., 2013; Xin et al., 2013). A DNA barcoding system for herbal medicine, which is based on ITS2 as the core DNA barcode, has been established. An online DNA barcoding database has also been constructed (http://www.tcmbarcode.cn) (Chen et al., 2014). The principle for the DNA barcoding of traditional Chinese medicine has been added to Supplement 3 of the Chinese Pharmacopeia (2010 edition). Therefore, the ITS2 barcode provides a suitable tool to identify traditional Chinese medicine and address problems regarding substitutes and adulterants.

### ITS2 barcode effectively identifies acanthopanacis cortex

Acanthopanacis cortex is increasingly becoming popular in clinical applications. However, several closely related species of *E. nodiflorus*, such as *E. giraldii, E. senticosus*, and *E. sessiliflorus*, are locally used as Acanthopanacis cortex in several places (Wang et al., 2005; Song et al., 2012). Moreover, Acanthopanacis cortex is seriously mistaken for Periplocae cortex when used in Chinese patent medicines or health supplement products (Guo et al., 2013). Existing toxic adulterants are important factors causing safety issues. Thus, correct identification of Acanthopanacis cortex is absolutely essential to ensure clinical safety. Traditional identification methods cannot easily authenticate naturally dried, sliced, shredded, or simply processed herbal medicine. Morphological authentication approach largely depends on taxonomists and becomes infeasible because of the absence of identifying features. The ITS2 barcode offers the potential to resolve this problem. Liu et al. (2012) indicated that ITS2 can be easily used for PCR amplification and sequencing and is a powerful barcode for Araliaceae identification. Sun and Chen (2013)used ITS2 to identify cortex herbs listed in the Chinese Pharmacopeia; they found that genuine cortex herbs can be discriminated from false ones and also concluded that the ITS2 barcode is suitable to identify cortex herbs in the Chinese Pharmacopeia. The materials in the aforementioned studies comprised leaf tissues and sequences downloaded from GenBank. In the present research, the studied materials mainly included cortexes from medicine markets and drug stores. Adulterants of Acanthopanacis cortex were also analyzed. Genomic DNA from the cortex tissues was successfully acquired. The results of SNPs, nearest distance, and NJ tree methods indicated that the ITS2 barcode can successfully identify Acanthopanacis cortex and its adulterants. Among the adulterants, *E. senticosus* and *E. sessiliflorus* were distributed in the same areas and easily confused (Zhu et al., 2011). Our research demonstrated that ITS2 can also distinguish these two species from one another (Figure 2).

### DNA barcoding is a convenient tool for medicine market supervision

With the development of DNA barcoding, its application in identifying Chinese medicine has also expanded. In some studies, DNA barcoding was applied to supervise medicine markets. For instance, Xin et al. (2015) constructed a DNA barcode database with 82 voucher samples from 10 *Rhodiola* species and used this database to identify 100 Rhodiolae Crenulatae Radix et Rhizoma decoction samples from hospitals and drug stores; their results indicated that only 36 samples (40%) were authentic. Wu et al. (2015) established efficient method with DNA barcoding to identify Aristolochiaceous herbs; this method can protect consumers from health risks caused by aristolochic acids, which can lead to aristolochic acid nephropathy. In the current research, DNA barcoding was precisely verified to supervise medicine markets. We detected five adulterants by using the ITS2 barcode from all nine Acanthopanacis cortex samples purchased from drug stores and medicine markets (Supplementary Table 2). One of the five adulterants was derived from *E. giraldii*, also known as “hongmao-wujiapi,” which was reportedly used as Acanthopanacis cortex in several places (Wang et al., 2005). Previous studies have reported that *P. sepium*, also known as “Periplocae cortex,” was easily confused with Acanthopanacis cortex (Guo et al., 2013; Wu et al., 2014). In the present study, four out of the five adulterants were derived from *P. sepium* (Supplementary Table 2). We concluded that Periplocae cortex is a common adulterant of Acanthopanacis cortex in medicine markets and drug stores. Two main reasons can explain why the adulterant Periplocae cortex is common in medicine markets. First, Acanthopanacis cortex and Periplocae cortex are similar in morphological characteristics and difficult to distinguish from each other (Figure 1). Second, Acanthopanacis cortex and Periplocae cortex always use the names “Nanwujiapi” and “Beiwujiapi” in medicine markets, respectively. These two names sound similar and can be easily confused. Hence, we suggest that the ITS2 barcode should be used to monitor Acanthopanacis cortex, and the name “Xiangjiapi” should be used instead of “Beiwujiapi” in medicine markets.

### Conflict of interest statement

The authors declare that the research was conducted in the absence of any commercial or financial relationships that could be construed as a potential conflict of interest.

## Supplementary material

Supplementary Table 1 **Plant materials used in this study**.

Supplementary Table 2 **Authenticity of commercial samples of Acanthopanacis cortex collected from medicine markets and drug stores**.

Supplementary Figure 1 **Variable sites in haplotypes of the five *Eleutherococcus* species**.
