# Supplementary material for: Internal transcribed spacer 2 barcode: a good tool for identifying Acanthopanacis cortex
Source: Front Plant Sci. 2015 Oct 8;6:840. doi: 10.3389/fpls.2015.00840 (PMC4597102; doi:10.3389/fpls.2015.00840)
Supplement: Supplementary Table 1 — Plant materials used in this study. [file Table1.DOCX]

**Supplementary Table 1. Plant materials used in this study**

| Species | Voucher no. | GenBank no. | Collection place | Collection tissiue |
| --- | --- | --- | --- | --- |
| *Eleutherococcus nodiflorus* (Dunn) S.Y.Hu | YC0123MT02 | KR080394 | Institute of Medicinal Plant Development, Beijing | Cortex |
| *E. nodiflorus* | YC0123MT14 | KR080395 | Drug store, Shijiazhuang, Hebei | Cortex |
| *E. nodiflorus* | YC0123MT15 | KR080396 | Drug store, Beijing | Cortex |
| *E. nodiflorus* | YC0123MT16 | KR080397 | Drug store, Tianjin | Cortex |
| *E. nodiflorus* | YC0123MT17 | KR080398 | Drug store, Wuhan, Hubei | Cortex |
| *E. nodiflorus* | YC0123MT18 | KR080399 | Medicinal Plant Garden,  Guangdong Pharmaceutical University | Cortex |
| *E. nodiflorus* | YC0123MT20 | KR080400 | National Institutes for Food and Drug Control, Beijing | Cortex |
| *E. nodiflorus* | PS1473MT01 | GQ434779 | Institute of Medicinal Plant Development, Beijing | Leaf |
| *E. nodiflorus* | YC0123MT06 | KR080401 | Lushan, Jiangxi | Leaf |
| *E. nodiflorus* | YC0123MT07 | KR080402 | Lushan, Jiangxi | Leaf |
| *E. nodiflorus* | YC0123MT08 | KR080403 | Lushan, Jiangxi | Leaf |
| *E. nodiflorus* | YC0123MT09 | KR080404 | Wuhan Botanical Garden, Hubei | Leaf |
| *E. nodiflorus* | YC0123MT10 | KR080405 | Wuhan Botanical Garden, Hubei | Leaf |
| *E. nodiflorus* | YC0123MT11 | KR080406 | Wuhan Botanical Garden, Hubei | Leaf |
| *E. nodiflorus* | YC0123MT12 | KR080407 | Lushan, Jiangxi | Leaf |
| *E. nodiflorus* | YC0123MT13 | KR080408 | Nanyang, Henan | Leaf |
| *Periploca* *sepium* Bge | YC0116MT01 | KR080409 | Hehuachi Medicine Market, Chengdu, Sichuan | Cortex |
| *P. sepium* | YC0116MT02 | KR080410 | Anguo Medicine Market, Baoding, Hebei | Cortex |
| *P. sepium* | YC0116MT06 | KR080411 | Drug store, Beijing | Cortex |
| *P. sepium* | YC0116MT07 | KR080412 | National Institutes for Food and Drug Control, Beijing | Cortex |
| *P. sepium* | YC0116MT08 | KR080413 | Drug store, Tianjin | Cortex |
| *P. sepium* | YC0116MT09 | KR080414 | Drug store, Kunming, Yunnan | Cortex |
| *P. sepium* | YC0116MT10 | KR080415 | Hospital pharmacy, Beijing | Cortex |
| *P. sepium* | YC0116MT12 | KR080416 | Yulin Medicine Market, Yulin, Guangxi | Cortex |
| *P. sepium* | YC0116MT15 | KR080417 | Drug store, Beijing | Cortex |
| *P. sepium* | YC0116MT17 | KR080418 | Bozhou Medicine Market, Bozhou, Anhui | Cortex |
| *P. sepium* | YC0116MT20 | KR080419 | Hehuachi Medicine Market, Chengdu, Sichuan | Cortex |
| *P. sepium* | YC0116MT21 | KR080420 | Drug store, Beijing | Cortex |
| *P. sepium* | YC0116MT13 | KR080421 | Nanchuan, Chongqing | Leaf |
| *P. sepium* | YC0116MT14 | KR080422 | Nanchuan, Chongqing | Leaf |
| *P. sepium* | PS0840MT01 | GQ434573 | Institute of Medicinal Plant Development, Beijing | Leaf |
| *Eleutherococcus senticosus* Maxim. | YC0465MT08 | KR080423 | Anguo Medicine Market, Baoding, Hebei | Radix |
| *E. senticosus* | YC0465MT09 | KR080424 | Anguo Medicine Market, Baoding, Hebei | Radix |
| *E. senticosus* | YC0465MT11 | KR080425 | Bozhou Medicine Market, Bozhou, Anhui | Radix |
| *E. senticosus* | YC0465MT12 | KR080426 | Bozhou Medicine Market, Bozhou, Anhui | Radix |
| *E. senticosus* | YC0465MT20 | KR080427 | Drug store, Beijing | Radix |
| *E. senticosus* | YC0465MT23 | KR080428 | Drug store, Beijing | Radix |
| *E. senticosus* | YC0465MT26 | KR080429 | Drug store, Beijing | Radix |
| *E. senticosus* | YC0465MT27 | KR080430 | Drug store, Beijing | Radix |
| *E. senticosus* | YC0465MT28 | KR080431 | Hospital pharmacy, Beijing | Radix |
| *E. senticosus* | YC0465MT29 | KR080432 | Hospital pharmacy, Beijing | Radix |
| *E. senticosus* | YC0465MT13 | KR080433 | Xi’an, Shaanxi | Stem |
| *E. senticosus* | YC0465MT19 | KR080434 | Drug store, Beijing | Fruit |
| *E. senticosus* | YC0465MT30 | KR080435 | Baihuashan, Beijing | Leaf |
| *E. senticosus* | CBS448MT01 | KR080436 | Changbaishan, Jilin | Leaf |
| *E. senticosus* | YC0465MT07 | KR080437 | Donglingshan, Beijing | Leaf |
| *Eleutherococcus giraldii* (Harms) Nakai | YC0802MT01 | KR080438 | Hehuachi Medicine Market, Chengdu, Sichuan | Cortex |
| *E. giraldii* | YC0802MT02 | KR080439 | Songpan, Sichuan | Cortex |
| *E. giraldii* | YC0802MT03 | KR080440 | Drug store, Guangzhou, Guangdong | Cortex |
| *E. giraldii* | YC0802MT04 | KR080441 | Chuqimen Medicine Market, Chongqing | Cortex |
| *E. giraldii* | YC0802MT05 | KR080442 | Yulin Medicine Market, Yulin, Guangxi | Cortex |
| *E. giraldii* | PS1461MT01 | GQ434776 | Maerkang, Sichuan | Leaf |
| *E. giraldii* | YC0802MT07 | KR080443 | Songpan, Sichuan | Leaf |
| *E. giraldii* | YC0802MT08 | KR080444 | Songpan, Sichuan | Leaf |
| *E. giraldii* | YC0802MT09 | KR080445 | Songpan, Sichuan | Leaf |
| *Eleutherococcus sessiliflorus* (Rupr. & Maxim.) S.Y.Hu | YC0494MT04 | KR080450 | Tonghua, Jilin | Stem |
| *E. sessiliflorus* | YC0494MT01 | KR080447 | Tonghua, Jilin | Leaf |
| *E. sessiliflorus* | YC0494MT02 | KR080448 | Tonghua, Jilin | Leaf |
| *E. sessiliflorus* | YC0494MT03 | KR080449 | Tonghua, Jilin | Leaf |
| *E. sessiliflorus* | YC0494MT05 | KR080451 | Tonghua, Jilin | Leaf |
| *E. sessiliflorus* | PS1471MT01 | GQ434778 | Institute of Medicinal Plant Development, Beijing | Leaf |
| *E. sessiliflorus* | CBS429MT01 | KR080452 | Changbaishan, Jilin | Leaf |
| *E. sessiliflorus* | CBS429MT02 | KR080453 | Changbaishan, Jilin | Leaf |
| *E. sessiliflorus* | CBS429MT03 | KR080454 | Changbaishan, Jilin | Leaf |
| *Eleutherococcus trifoliatus* (L.) S.Y.Hu | YC0823MT01 | KR080446 | Medicinal Plant Garden,  Guangdong Pharmaceutical University | Leaf |
| *E. trifoliatus* | — | AF551739 | GenBank |  |
| *E. trifoliatus* | — | AY548189 | GenBank |  |
| *E. trifoliatus* | — | AY725119 | GenBank |  |
| *E. trifoliatus* | — | DQ007373 | GenBank |  |
