# Supplementary material for: Internal transcribed spacer 2 barcode: a good tool for identifying Acanthopanacis cortex
Source: Front Plant Sci. 2015 Oct 8;6:840. doi: 10.3389/fpls.2015.00840 (PMC4597102; doi:10.3389/fpls.2015.00840)
Supplement: Supplementary Table 2 — Authenticity of commercial samples of Acanthopanacis cortex collected from medicine markets and drug stores. [file Table2.DOCX]

**Supplementary Table 2. Authenticity of commercial samples of Acanthopanacis cortex collected from medicine markets and drug stores.**

| Label name | Identified Species | Authenticity (Yes or No) | Voucher no. | Collection place |
| --- | --- | --- | --- | --- |
| *Eleutherococcus nodiflorus* (Dunn) S.Y.Hu | *Eleutherococcus nodiflorus* (Dunn) S.Y.Hu | Yes | YC0123MT14 | Drug store, Shijiazhuang, Hebei |
| *E. nodiflorus* | *E. nodiflorus* | Yes | YC0123MT15 | Drug store, Beijing |
| *E. nodiflorus* | *E. nodiflorus* | Yes | YC0123MT16 | Drug store, Tianjin |
| *E. nodiflorus* | *E. nodiflorus* | Yes | YC0123MT17 | Drug store, Wuhan, Hubei |
| *E. nodiflorus* | *Periploca* *sepium* Bge | No | YC0116MT15 | Drug store, Beijing |
| *E. nodiflorus* | *P. sepium* | No | YC0116MT17 | Bozhou Medicine Market, Bozhou, Anhui |
| *E. nodiflorus* | *P. sepium* | No | YC0116MT20 | Hehuachi Medicine Market, Chengdu, Sichuan |
| *E. nodiflorus* | *P. sepium* | No | YC0116MT21 | Drug store, Beijing |
| *E. nodiflorus* | *Eleutherococcus giraldii* (Harms) Nakai | No | YC0802MT01 | Hehuachi Medicine Market, Chengdu, Sichuan |
