# Supplementary figures and images for: Internal transcribed spacer 2 barcode: a good tool for identifying Acanthopanacis cortex
Source: Front Plant Sci. 2015 Oct 8;6:840. doi: 10.3389/fpls.2015.00840 (PMC4597102; doi:10.3389/fpls.2015.00840)

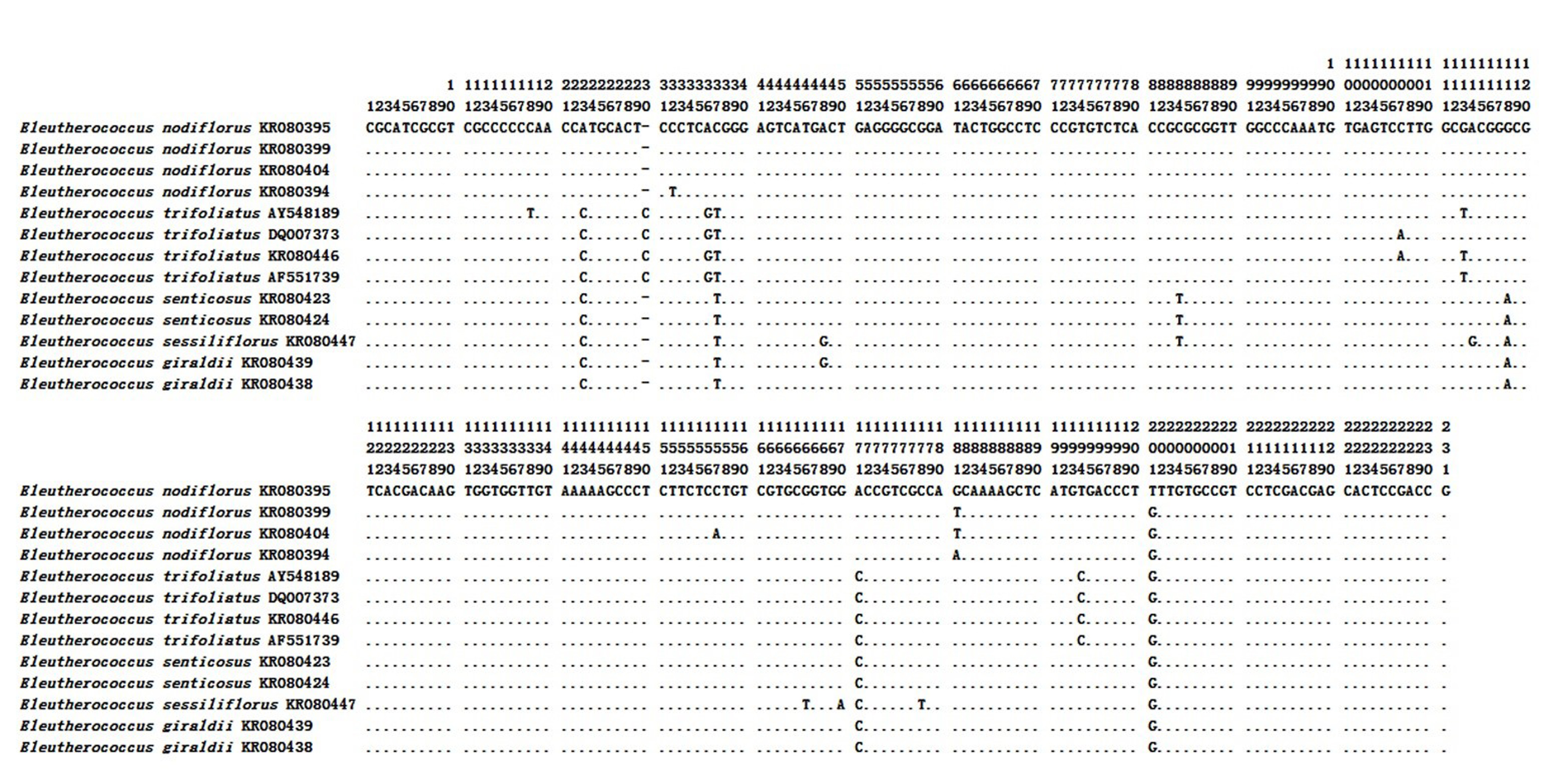

Supplement: Supplementary Figure 1 — Variable sites in haplotypes of the five Eleutherococcus species. [file Image1.JPEG]
